# *QuickStats:* Age-Adjusted Percentage* of Adults Aged ≥25 Years Who Saw a Dentist in the Past Year,**^†^** by Education Level and Sex — National Health Interview Survey,**^§^** 2018

**DOI:** 10.15585/mmwr.mm6907a6

**Published:** 2020-02-21

**Figure:**
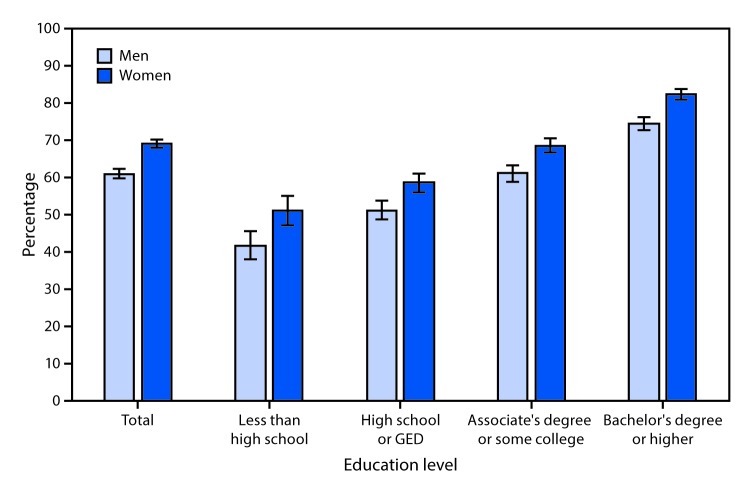
In 2018, among adults aged ≥25 years, women (69.4%) were more likely than men (61.2%) to have seen a dentist in the past year. The percentage of men and women who saw a dentist in the past year increased as education level increased. Among women, those with a Bachelor’s degree or higher were the most likely to have seen a dentist in the past year (82.5%) and those with less than a high school education were least likely (51.4%). Among men, the same pattern prevailed (74.6% compared with 41.9%). Within each education group, the percentage of women who saw a dentist in the past year was higher than the percentage of men.

**Source:** National Health Interview Survey, 2018. https://www.cdc.gov/nchs/nhis/index.htm.

